# Supplementary figures and images for: Optimizing Cost-Effective gene expression phenotyping approaches in cattle using 3′ mRNA sequencing
Source: BMC Genomics. 2025 Apr 16;26:379. doi: 10.1186/s12864-025-11571-4 (PMC12001630; doi:10.1186/s12864-025-11571-4)

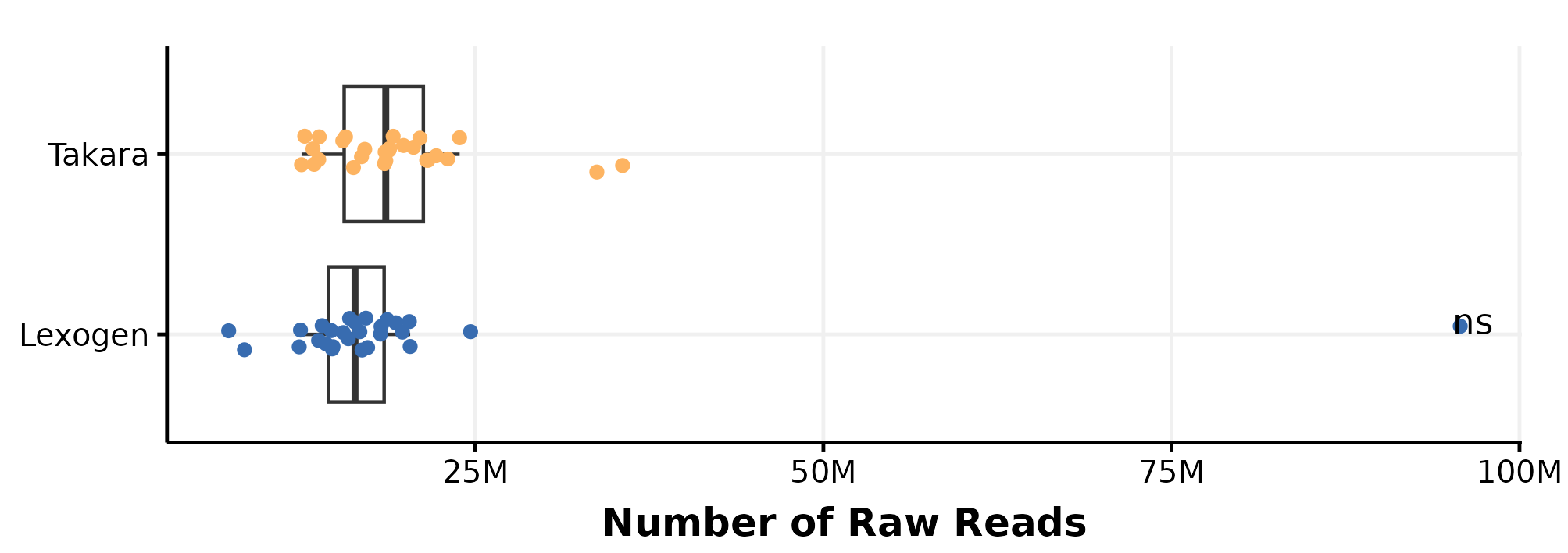

Supplement: Supplementary file 1 — Supplementary Material 1 [file 12864_2025_11571_MOESM1_ESM.png]

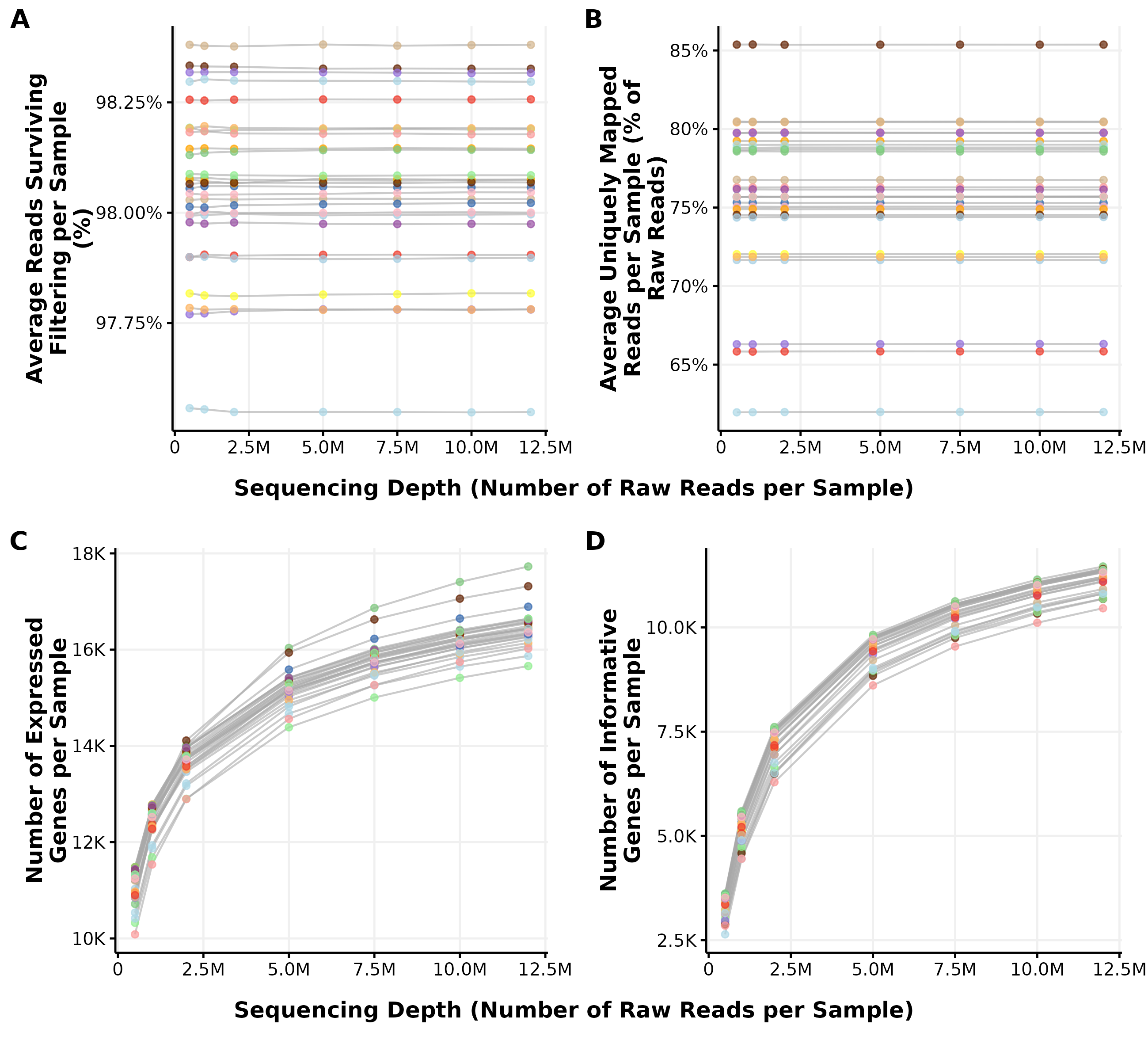

Supplement: Supplementary file 2 — Supplementary Material 2 [file 12864_2025_11571_MOESM2_ESM.png]

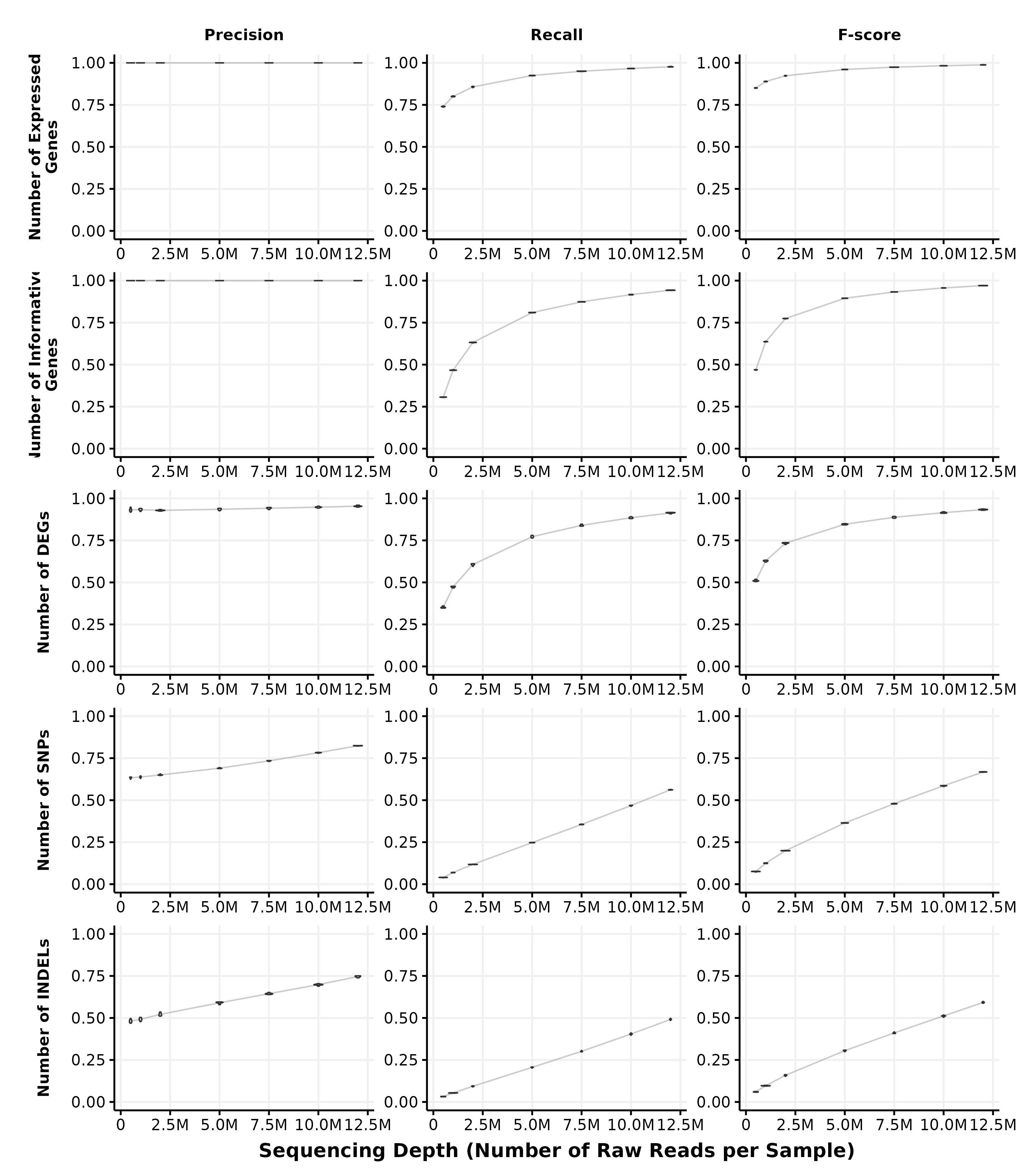

Supplement: Supplementary file 3 — Supplementary Material 3 [file 12864_2025_11571_MOESM3_ESM.png]

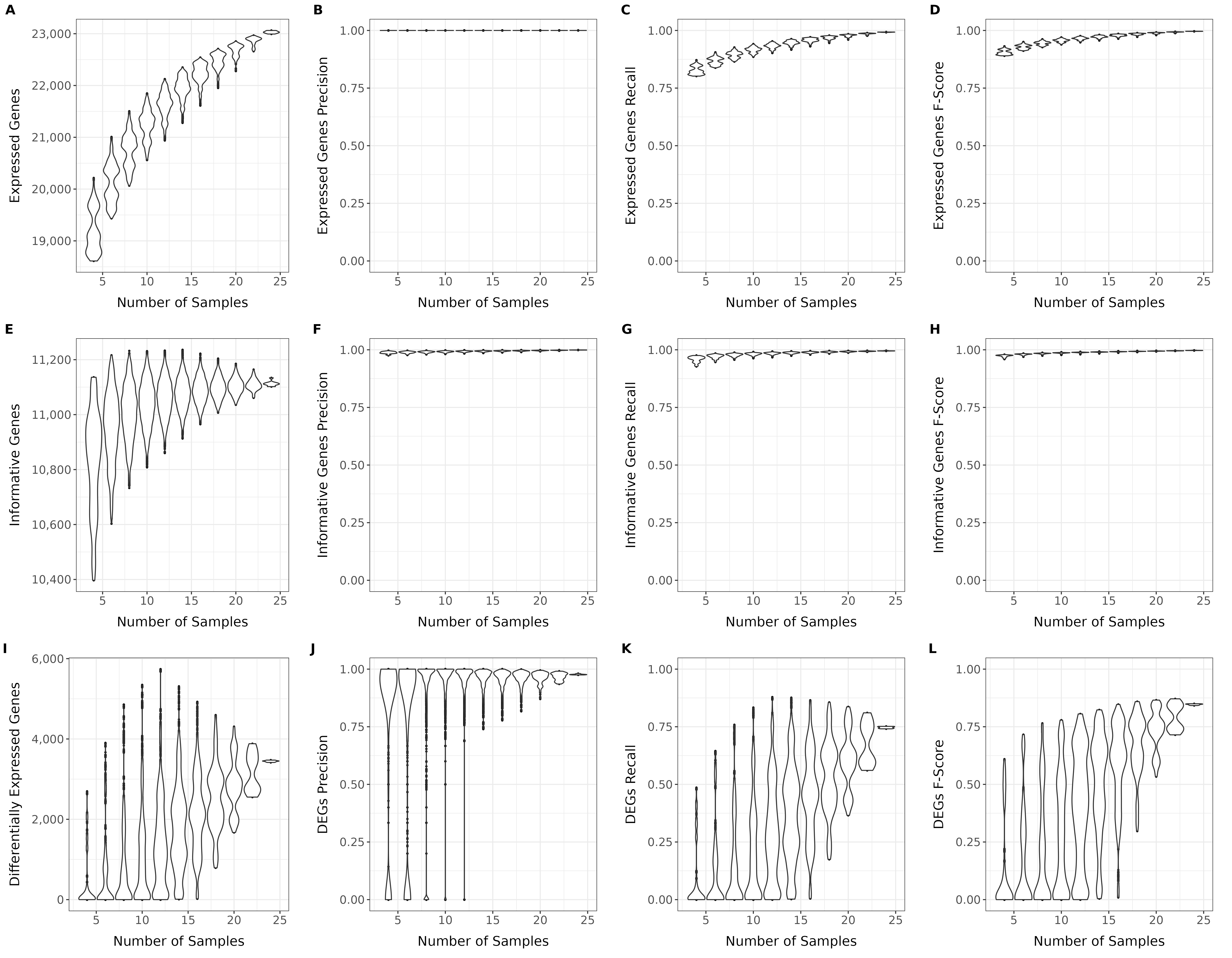

Supplement: Supplementary file 4 — Supplementary Material 4 [file 12864_2025_11571_MOESM4_ESM.png]
