# Supplementary material for: Optimizing Cost-Effective gene expression phenotyping approaches in cattle using 3′ mRNA sequencing
Source: BMC Genomics. 2025 Apr 16;26:379. doi: 10.1186/s12864-025-11571-4 (PMC12001630; doi:10.1186/s12864-025-11571-4)
Supplement: Supplementary file 5 — Supplementary Material 5 [file 12864_2025_11571_MOESM5_ESM.docx]

Optimizing Cost-Effective Gene Expression Phenotyping Approaches in Cattle Using 3*′* mRNA Sequencing

Ruwaa I. Mohamed^1^, Taylor B. Ault-Seay^2^, Sonia Moisa^2^, Jonathan Beever^1,2^, Agustín G. Ríus ^2^, Troy Rowan^1,2,*^

^1^Genome Science and Technology Program, Bredesen Center, University of Tennessee, Knoxville, TN, USA.

^2^Animal Science Department, University of Tennessee Institute of Agriculture (UTIA), Knoxville, TN, USA.

* Corresponding author.

# Supplementary Figures


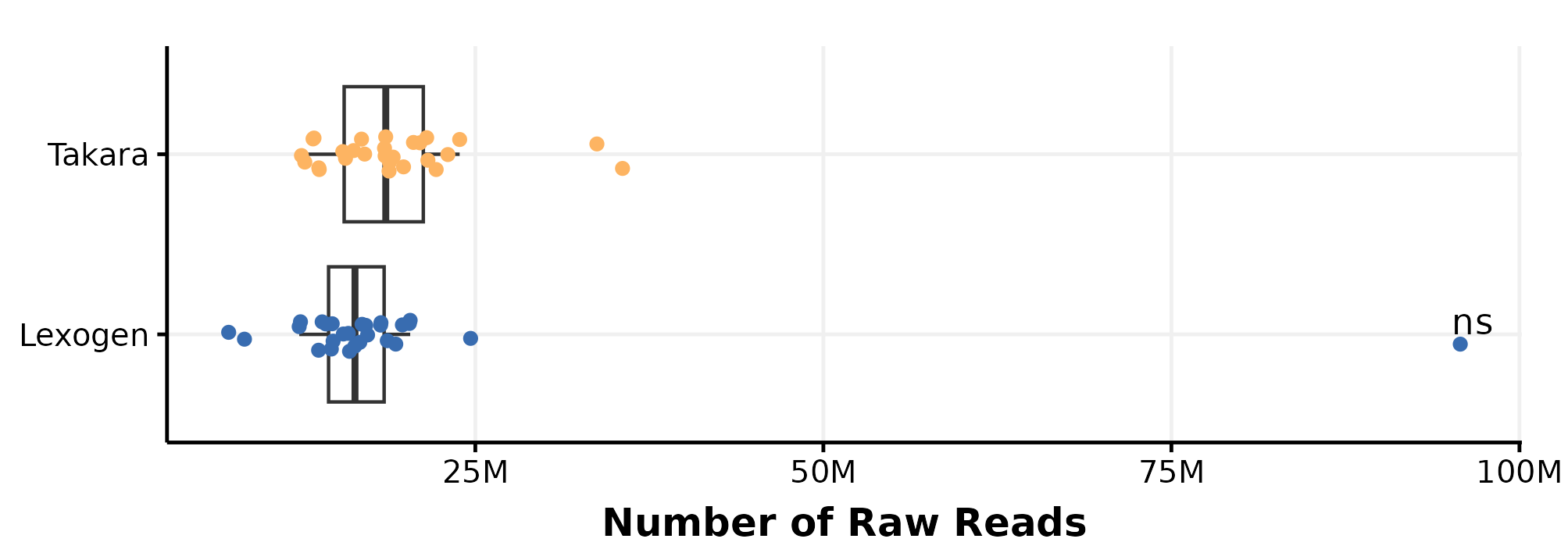


***Figure S1:*** *Number of raw reads obtained from sequencing with Takara and Lexogen 3′ mRNA-Seq library kits (p-value = 0.9761). We obtained a median of 18.6 M (range: 12.5 M - 35.6 M) from Takara libraries (top) and 16.4 M (range: 7.3 M - 95.7 M) raw reads per sample from Lexogen libraries (bottom).*

*
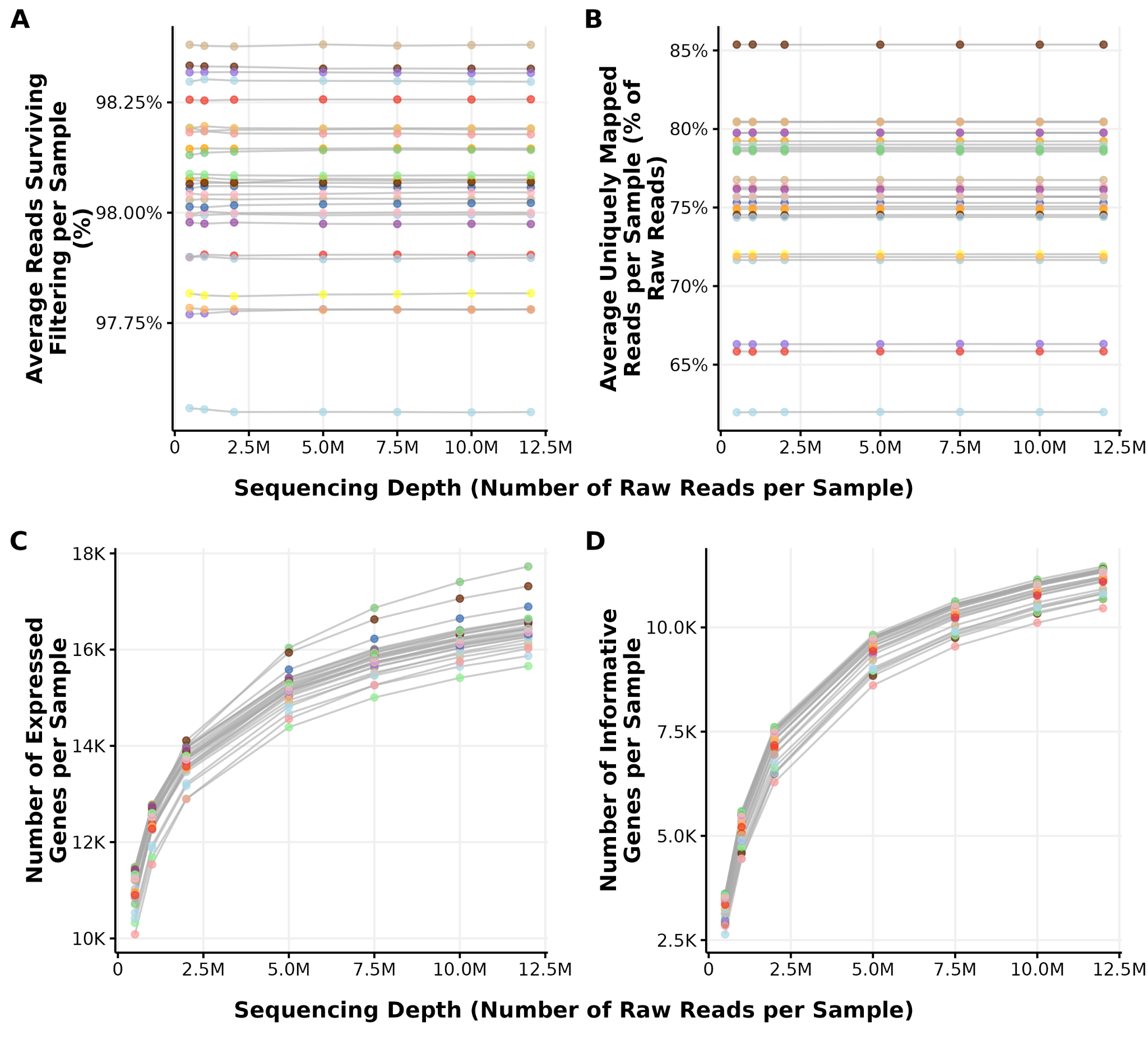
*

***Figure S2:*** *Effect of sequencing depth for Takara libraries on per-sample metrics. Each line represents a sample, and each dot is the average of ten replicates. Each color represents one animal. (A) Average read filtration rate (%) as a function of the number of raw reads per sample. (B) Per sample average uniquely mapped read (% of raw reads) as a function of the number of raw reads per sample. Both the filtering rate and mapping rate are entirely dependent on the sample and the quality of the sample (handling, extraction protocol, library preparation, sequencer, etc.) and independent of the sequencing depth. (C. D) Number of expressed genes (C) and informative genes (D) per sample as a function of sequencing depth (Number of raw reads per sample). Both metrics are dependent on the sequencing depth, similar to Figure 6.*

*
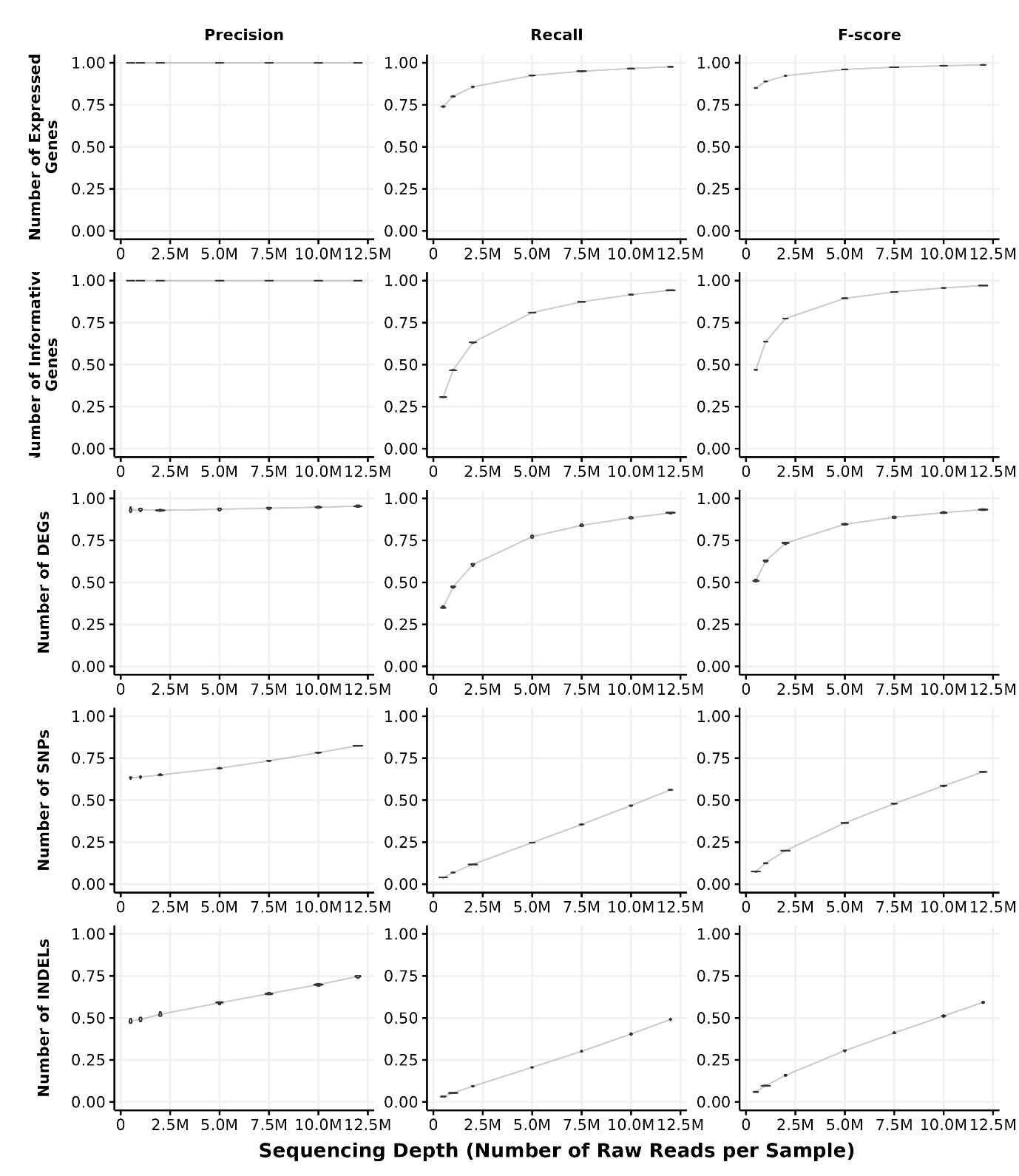
*

***Figure S3:*** *Precision (first column), Recall (second column), and F-score (third column) for expressed genes (first row), informative genes (second row), differentially expressed genes (DEGs)(third row), SNPs (fourth row), and INDELS (fifth row) as a function of sequencing depth (number of reads per sample).*

*
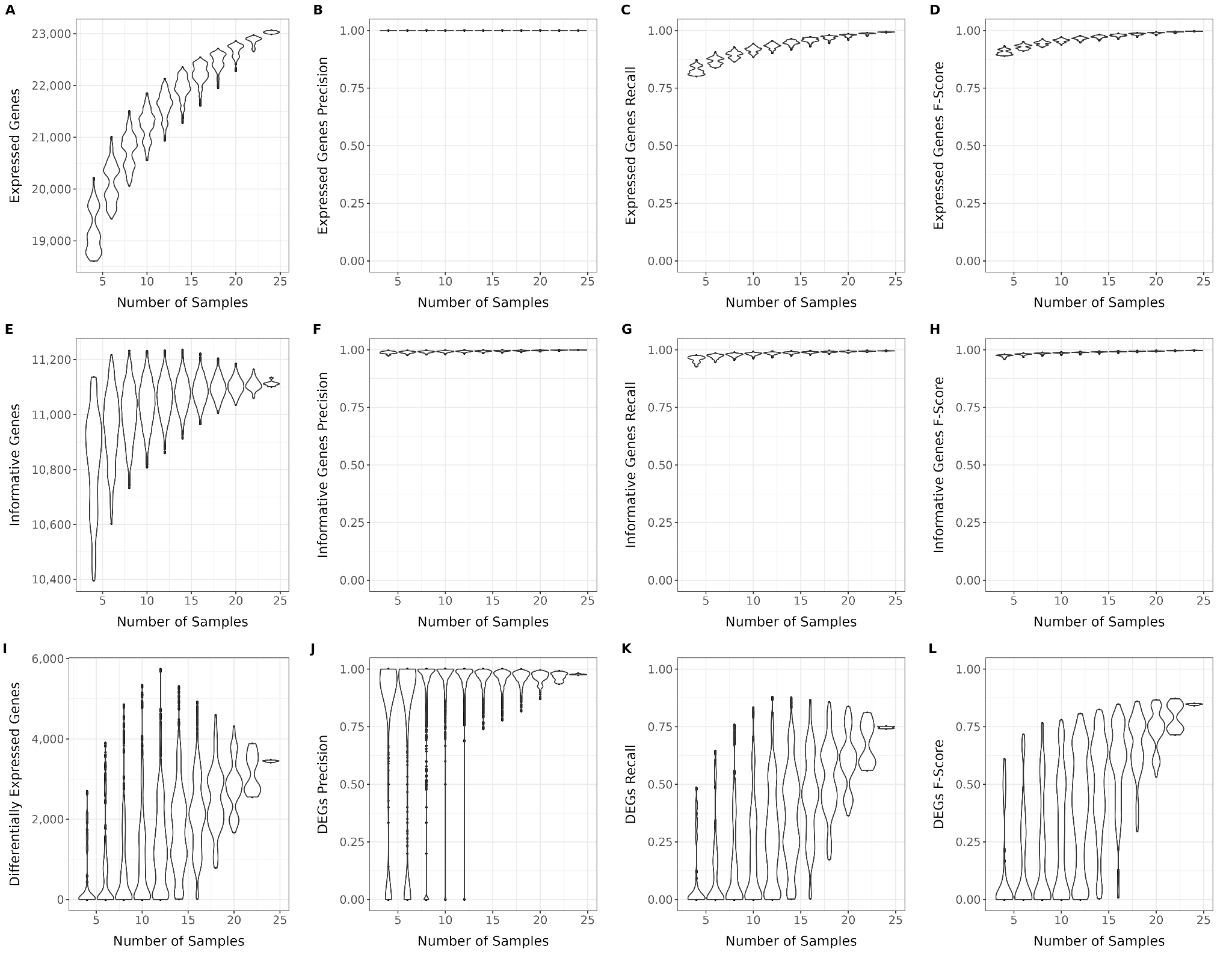
*

***Figure S4****: Effect of sample size on the number of expressed genes (A-D), informative genes (E-H), and differentially expressed genes (I-L).*

# Supplementary Tables

***Table S1:*** *Study design of the 3′ mRNA-Seq libraries used to sequence 29 biological samples representing 15 calves before and after exposure to heat stress for 12 hours. There are 27 samples sequenced with the Takara library and 27 samples sequences with the Lexogen library.*

| Calf | Thermoneutral control | Heat stress |
| --- | --- | --- |
| 1 | Takara & Lexogen | Takara & Lexogen |
| 2 | Takara | Takara & Lexogen |
| 3 | Takara & Lexogen | Takara & Lexogen |
| 4 | Takara & Lexogen | Takara & Lexogen |
| 5 | Takara & Lexogen | Takara |
| 6 | Takara & Lexogen | Takara & Lexogen |
| 7 | Takara & Lexogen | Takara & Lexogen |
| 8 | Takara & Lexogen | Takara & Lexogen |
| 9 | Takara & Lexogen | --- |
| 10 | Lexogen | Takara & Lexogen |
| 11 | Takara & Lexogen | Lexogen |
| 12 | Takara & Lexogen | Takara & Lexogen |
| 13 | Takara & Lexogen | Takara & Lexogen |
| 14 | Takara & Lexogen | Takara & Lexogen |
| 15 | Takara & Lexogen | Takara & Lexogen |
